# Gene expression profiles associated with stimulus-responsive insulin secretion in insulinoma

**DOI:** 10.1530/EC-26-0322

**Published:** 2026-07-15

**Authors:** Kenji Sugawara, Yuko Okada, Tomokazu Matsuda, Yushi Hirota, Shun-ichiro Asahara, Kazuhiko Sakaguchi, Wataru Ogawa

**Affiliations:** ^1^ Division of Diabetes and Endocrinology, Department of Internal Medicine, Kobe University Graduate School of Medicine, Kobe, Japan; ^2^ Kagayaki Diabetes and Endocrinology Clinic Sannomiya, Kobe, Japan; ^3^ Matsuda Diabetes Clinic, Kobe, Japan; ^4^ Division of Community Medicine and Medical Education, Department of Social/Community Medicine and Health Science, Kobe University Graduate School of Medicine, Kobe, Japan; ^5^ Division of Metabolic Diseases, Department of Translational Medical Science, Kobe University Graduate School of Medicine, Kobe, Japan

**Keywords:** insulinoma, DNA microarray, hypoglycemia, insulin secretion, postprandial hypoglycemia

## Abstract

Insulinoma is a functional pancreatic neuroendocrine tumor that usually causes fasting hypoglycemia through inappropriate autonomous insulin secretion. However, some insulinomas show postprandial or stimulus-induced hypoglycemia, suggesting that some tumors retain the ability to respond to physiological stimuli. We examined four patients with confirmed insulinoma who showed different clinical patterns of hypoglycemia and integrated clinical stimulation tests with DNA microarray analysis. Insulin secretory dynamics were assessed using an oral glucose tolerance test, meal tolerance test, and glucagon stimulation test, as clinically indicated. Among the four cases, Case 1 showed no clear fasting hypoglycemia but developed hypoglycemia after meals and oral glucose loading, accompanied by marked insulin secretion. In this case, insulin or C-peptide increased markedly after glucose, meal, and glucagon stimulation. In contrast, the other three cases showed relatively weak insulin secretory responses to stimulation. During the fasting test, Cases 3 and 4 developed hypoglycemia early, whereas Case 2 showed a prolonged time to hypoglycemia. Transcriptomic analysis showed that, by hierarchical clustering, Case 1 was clearly separated from Cases 3 and 4, whereas Case 2 was relatively close to Case 1. Case 1 showed relatively preserved expression of genes involved in glucose sensing, ATP-sensitive potassium channel function, incretin/cAMP signaling, exocytosis, and β-cell differentiation. In contrast, cases with predominant fasting hypoglycemia showed higher expression of hexokinase 1 and stress-response genes. These findings suggest that clinical heterogeneity in insulinoma may reflect differences in β-cell-like stimulus-response mechanisms, glucose sensing, stress responses, and differentiation status.

## Introduction

Insulinoma is a functional pancreatic neuroendocrine tumor that causes endogenous hyperinsulinemic hypoglycemia (1, 2). In typical cases, insulin secretion remains inappropriately elevated despite low blood glucose, leading to fasting hypoglycemia, early-morning neuroglycopenic symptoms, impaired consciousness, or seizures. However, insulinomas are functionally heterogeneous, and some patients develop hypoglycemia predominantly after meals or oral glucose loading.

Previous functional studies of human insulinomas have shown that insulin secretory patterns vary substantially among tumors (3). These studies also suggest that histologic appearance alone cannot fully explain the secretory behavior of insulinoma. Therefore, molecular evaluation of pathways related to insulin secretion, glucose sensing, exocytosis, and β-cell differentiation may provide additional insights into the clinical heterogeneity of insulinoma.

Glucose-stimulated insulin secretion in β-cells depends on coordinated glucose sensing, K_ATP_ channel activity, incretin/cAMP signaling, and exocytosis of insulin granules (4, 5, 6, 7, 8). Therefore, expression of genes involved in these pathways may provide insights into the mechanisms underlying stimulus-responsive insulin secretion in insulinoma.

In this study, we compared four insulinoma cases with different clinical patterns of hypoglycemia. We integrated clinical stimulation tests and tumor gene expression profiling to explore molecular features associated with stimulus-responsive insulin secretion in insulinoma.

## Materials and methods

### Patients and clinical evaluation

Four patients who underwent surgical treatment for insulinoma at a university hospital and whose tumor tissues were subjected to DNA microarray analysis were included in this retrospective study. The clinical diagnosis of insulinoma was established on the basis of clinical hypoglycemia with inappropriate insulin secretion, biochemical findings, imaging studies, and selective arterial calcium stimulation testing (SACST) when clinically indicated. Surgical resection was performed in all patients, and the diagnosis was confirmed pathologically after surgery. Clinical information, including symptoms, imaging findings, biochemical data, and stimulation test results, was retrospectively reviewed. Pathological findings, including tumor size, Ki-67 index, and WHO grade, were reviewed from the pathological reports. SACST was performed as part of the clinical evaluation for tumor localization, and the maximum insulin response after calcium injection was recorded for each case. The study was conducted in accordance with the Declaration of Helsinki and was approved by the Ethics Committee of Kobe University Hospital (Approval Number: 79).

### Stimulation tests

To evaluate insulin secretory dynamics, 75-g oral glucose tolerance tests, meal tolerance tests, and glucagon stimulation tests were performed as clinically indicated. Blood glucose, plasma immunoreactive insulin (IRI), and C-peptide were measured before and after stimulation. During the 75-g oral glucose tolerance test, samples were obtained at 0, 30, 60, 90, and 120 min. During the meal tolerance test, samples were obtained at 0, 60, 120, 180, 240, and 300 min. During the glucagon stimulation test, samples were obtained before and 6 min after glucagon administration.

### DNA microarray analysis

Total RNA extracted from surgically resected insulinoma tumor tissue was subjected to DNA microarray analysis by DNA Chip Research Inc. (Japan). Tumor tissues were snap-frozen in liquid nitrogen immediately after surgical enucleation and stored at −80°C until RNA extraction. RNA quality was assessed before microarray analysis, and the RNA integrity number values were 8.7, 8.1, 7.7, and 5.7 for Cases 1–4, respectively. Gene expression profiling was performed using the Agilent standard one-color method with an 8 × 60 K human gene expression microarray format. Briefly, 100 ng of total RNA from each sample were used for cRNA synthesis and Cy3 labeling according to the standard Agilent protocol. Hybridized arrays were scanned using an Agilent SureScan G4900DA microarray scanner, and signal intensities were extracted using Agilent Feature Extraction software (version 11.5.1.1).

### Data analysis

Normalized signal intensities were used for gene expression analysis. Probes judged as detected in at least one of the four cases were retained, yielding 33,018 probes and 22,086 unique genes. All analyses were performed at the probe level. Multiple probes corresponding to the same gene symbol were not collapsed into a single gene-level value. The reported number of unique genes indicates the number of gene symbols represented among the retained probes. For heatmap analysis of all genes, log2-transformed normalized expression values were converted to probe-level z-scores. Hierarchical clustering was performed using Pearson correlation distance and average linkage. Heatmaps were displayed using a green–black–red scale, with red indicating relatively high expression and green indicating relatively low expression.

Gene expression data were initially normalized using GeneSpring software, version 13.1.1, with quantile normalization. Subsequent analyses, including MA plot analysis, heatmap generation, hierarchical clustering, and calculation of relative expression values, were performed using Python with pandas, NumPy, SciPy, and Matplotlib. Genes related to insulin secretion and β-cell development/differentiation were selected based on gene ontology annotations (9). The insulin secretion category included ‘insulin secretion’ and ‘regulation of insulin secretion’, together with their descendant terms. The β-cell development/differentiation category included ‘type B pancreatic cell development’, ‘type B pancreatic cell differentiation’, ‘type B pancreatic cell maturation’, ‘endocrine pancreas development’, and ‘pancreas development’, together with their descendant terms. Hierarchical clustering and heatmap analysis were performed using log2-transformed normalized expression values. For MA plot analysis, Case 1 was compared with the mean expression of Cases 2–4, and the mean expression intensity was plotted against the log2 fold change. Genes with a log2 fold change ≥1 were classified as upregulated, whereas genes with a log2 fold change ≤ −1 were classified as downregulated. For selected genes, relative expression values were calculated using the expression level in Case 1 as 1.0.

## Results

### Clinical characteristics

Clinical characteristics of the four patients are summarized in Table 1. The patients were 63–82 years of age and included two men and two women. BMI ranged from 20.7 to 33.3 kg/m^2^. Preoperative HbA1c values were 4.7, 5.7, 5.1, and 5.1% in Cases 1–4, respectively. Case 1 presented with postprandial disturbance of consciousness and showed no clear fasting hypoglycemia during the fasting test. In contrast, Cases 2–4 presented with symptoms suggestive of fasting hypoglycemia. During the 72-h fasting test, Cases 3 and 4 developed hypoglycemia early, at 5 and 4 h, respectively, whereas Case 2 developed hypoglycemia only after 34 h of fasting. These findings indicate that the clinical pattern of hypoglycemia differed among the four cases, with Case 1 showing a predominantly postprandial phenotype.

**Table 1:** Clinical characteristics of the four insulinoma cases.

		Case 1	Case 2	Case 3	Case 4
Age, years		81	69	82	63
Sex		Male	Male	Female	Female
BMI, kg/m^2^		20.7	23.9	33.3	21.4
HbA1c, %		4.7	5.7	5.1	5.1
Symptoms		Postprandial disturbance of consciousness	Early-morning disturbance of consciousness	Fasting hypoglycemic episodes	Fasting hypoglycemic episodes
Fasting hypoglycemia		−	+	+	+
Postprandial hypoglycemia		+	−	−	−
Hypoglycemia after glucose					
loading		+	−	−	−
Hypoglycemia after glucagon					
stimulation		+	−	N.T.	−
72-h fasting test					
Time to hypoglycemia		Not observed	34 h	5 h	4 h
Plasma glucose, mg/dL		68	37	50	42
IRI, μU/mL		2.0	6.0	7.0	15.0

BMI, body mass index; IRI, immunoreactive insulin; N.T., not tested; +, present; −, absent.

The pathological characteristics of the tumors are summarized in Table 2. Pathological examination showed that all four tumors were insulinomas with low proliferative activity and were classified as WHO grade 1. SACST was performed in all four cases and showed positive calcium-stimulated insulin responses consistent with tumor localization. The magnitude and responsible artery differed among cases. Detailed SACST findings are provided in Supplementary Table 1 (see section on Supplementary materials given at the end of the article).

**Table 2:** Pathological characteristics of the four insulinoma cases.

	Case 1	Case 2	Case 3	Case 4
Tumor location	Pancreatic head	Pancreatic head	Pancreatic body	Pancreatic head
Tumor size	20 × 10 mm	16 × 12 mm	12 × 11 mm	4 × 4 mm
Pathological diagnosis	Well-differentiated insulinoma	Well-differentiated insulinoma	Well-differentiated insulinoma	Insulinoma
Ki-67 index	1%	<1%	1%	<1%
WHO grade	G1	G1	G1	G1

### Insulin secretory dynamics during stimulation tests

**Figure 1:**
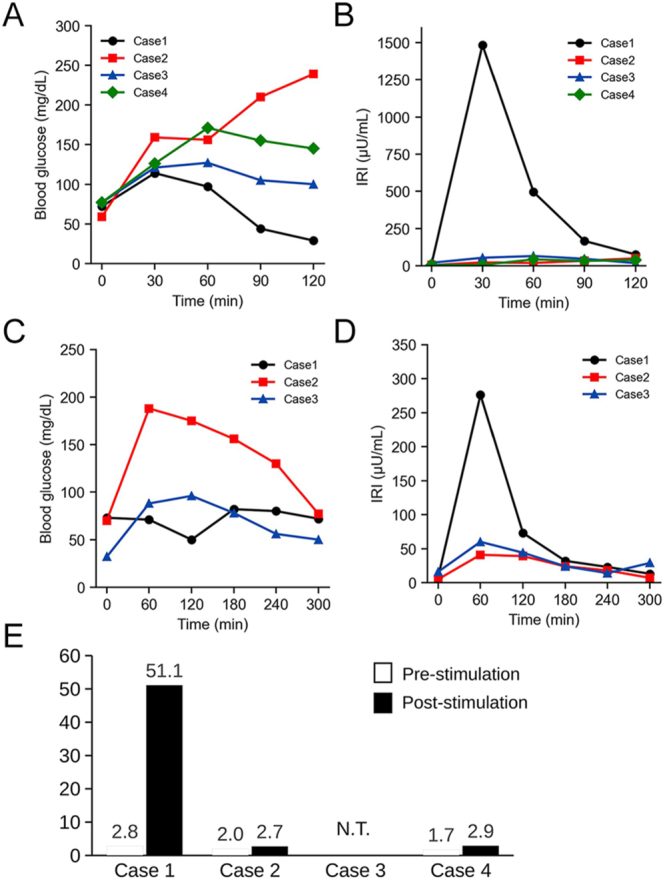
Insulin secretory dynamics during stimulation tests in four insulinoma cases. (A, B) Blood glucose and immunoreactive insulin (IRI) levels during the 75-g oral glucose tolerance test. (C, D) Blood glucose and IRI levels during the meal tolerance test. (E) C-peptide levels before and 6 min after glucagon stimulation. N.T., not tested.

The results of the stimulation tests are summarized in Fig. 1. Case 1 showed an exceptional stimulus-responsive pattern. During the 75-g oral glucose tolerance test, IRI increased to 1,483 μU/mL at 30 min, followed by a decrease in blood glucose. During the meal tolerance test, IRI increased to 276 μU/mL at 60 min, and postprandial hypoglycemia was observed. During the glucagon stimulation test, C-peptide increased from 1.8 ng/mL before stimulation to 51.1 ng/mL at 6 min.

In contrast, Cases 2–4 showed much weaker insulin or C-peptide responses during the 75-g oral glucose tolerance, meal, and glucagon stimulation tests than Case 1. Peak IRI during the oral glucose tolerance test was approximately 23, 66, and 43 μU/mL in Cases 2, 3, and 4, respectively. Post-load hypoglycemia was not observed in these cases. These findings suggest that Case 1 had markedly enhanced insulin secretory responsiveness to glucose, meal, and glucagon stimulation, whereas Cases 2–4 were characterized predominantly by autonomous insulin secretion during fasting.

Case 2 showed marked hyperglycemia during the preoperative OGTT, with a 2-h plasma glucose level of 239 mg/dL. However, the preoperative HbA1c level was 5.7%, and postoperative OGTT performed 15 days after tumor resection showed improvement in the 2-h plasma glucose level to 154 mg/dL, suggesting that established diabetes was unlikely. Detailed pre- and postoperative OGTT results for all cases are shown in Supplementary Table 2.

### Global transcriptomic patterns in insulinoma cases

**Figure 2:**
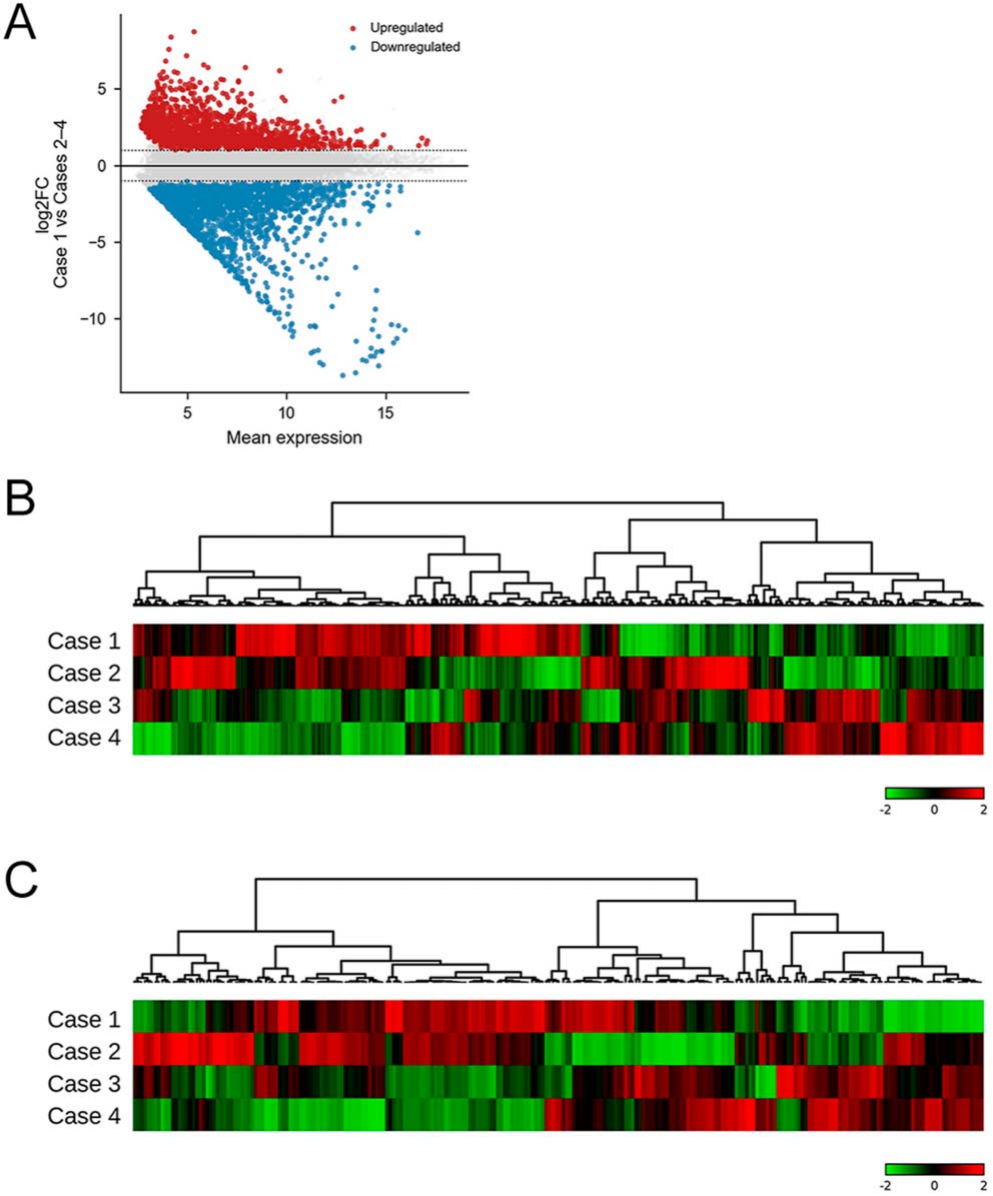
Gene expression patterns in insulinoma cases. DNA microarray analysis was used to compare gene expression patterns among the four cases. (A) MA plot comparing Case 1 with the mean of Cases 2–4. (B) Hierarchical clustering based on all detected genes. (C) Hierarchical clustering based on insulin secretion- and β-cell differentiation-related genes.

Tumor specimens obtained after surgical enucleation were subjected to DNA microarray analysis to compare global gene expression patterns among the four insulinoma cases. MA plot analysis was first performed to obtain an overview of gene expression differences between Case 1 and Cases 2–4. Compared with the mean expression level in Cases 2–4, Case 1 showed higher expression of 1,388 genes and lower expression of 1,672 genes (Fig. 2A), indicating broad transcriptomic differences between the stimulus-responsive case and the other cases.

Hierarchical clustering using all genes showed that Case 1 and Cases 3 and 4 formed distinct expression clusters. Case 2 did not completely match either Case 1 or Cases 3 and 4, but its overall expression pattern was relatively closer to Case 1 than to Cases 3 and 4 (Fig. 2B). When clustering was restricted to genes related to insulin secretion and β-cell differentiation, Case 1 and Cases 3 and 4 again showed distinct patterns, whereas Case 2 showed an intermediate pattern (Fig. 2C). These findings indicate that the stimulus-responsive phenotype of Case 1 was accompanied by differences in both global transcriptomic patterns and β-cell-related gene expression programs.

### Expression patterns of genes related to β-cell function

To explore candidate mechanisms underlying different secretory phenotypes, we selected representative genes related to β-cell function from the microarray dataset and grouped them into functional categories, including glucose sensing, K_ATP_ channel function, incretin/cAMP signaling, exocytosis, stress responses, and β-cell differentiation. Their expression levels were compared as relative values using Case 1 as the reference (Table 3).

**Table 3:** Relative expression of selected β-cell function-related genes.

Functional category	Gene	Case 1	Case 2	Case 3	Case 4
Glucose sensing/K_ATP_ channel	*ABCC8*	1	0.11	0.77	0.69
	*KCNJ11*	1	0.15	0.24	0.14
	*GCK*	1	0.56	0.31	0.16
	*HK1*	1	1.49	5.13	4.86
Incretin/cAMP signaling	*GIPR*	1	0.81	0.93	0.81
	*GLP1R*	1	0.27	0.37	0.52
	*PRKACA*	1	1.45	0.68	0.53
	*PRKACB*	1	0.24	0.49	0.48
	*PRKACG*	1	2.20	2.53	1
	*PRKCA*	1	0.32	0.60	0.67
	*RAPGEF4*	1	0.56	0.75	0.37
Exocytosis/vesicle trafficking	*RAB3A*	1	0.71	0.76	0.64
	*SNAP25*	1	2.03	1.53	0.81
	*SNAPIN*	1	0.76	0.74	0.67
	*SNX19*	1	0.54	0.40	0.40
	*STX1A*	1	0.69	0.34	0.31
	*SYT7*	1	0.09	0.40	0.36
Stress response/ER stress	*GDF15*	1	11.39	19.43	51.63
	*EIF2AK1*	1	0.78	0.68	0.61
	*EIF2AK2*	1	1.14	1.08	1.32
	*EIF2AK3*	1	1.88	2.06	2.45
	*EIF2AK4*	1	0.93	0.66	0.78
	*DDIT3*	1	1.16	0.96	0.54
	*ATF4*	1	2.62	2.04	2.25
	*XBP1*	1	6.32	6.23	6.73
β-cell differentiation/transcription factors	*HNF1A*	1	42.32	17.79	8.06
	*HNF1B*	1	0.51	1.64	2.69
	*HNF4A*	1	1.12	1.01	1.02
	*NEUROD1*	1	1.16	0.37	0.31
	*PAX6*	1	0.58	0.34	0.24
	*PDX1*	1	0.99	0.34	0.29

Values represent relative gene expression levels calculated using Case 1 as the reference. For each selected gene, the same probe was used consistently across all four cases. Gene symbols are shown in italics. K_ATP_, ATP-sensitive potassium; ER, endoplasmic reticulum.

Among genes involved in glucose sensing and K_ATP_ channel function, Case 1 showed relatively high expression of *ABCC8* and *KCNJ11*, which encode K_ATP_ channel components, and *GCK*, which encodes glucokinase. In Cases 2–4, relative expression values were 0.11–0.77 for *ABCC8*, 0.14–0.24 for *KCNJ11*, and 0.16–0.56 for *GCK*. In contrast, *HK1*, which encodes hexokinase 1, was expressed at higher levels in Cases 2–4, with relative values of 1.49–5.13, particularly in Cases 3 and 4.

For incretin and cAMP signaling-related genes, *GLP1R* expression was lower in Cases 2–4 than in Case 1, with relative values of 0.27–0.52. *GIPR* expression showed smaller inter-case differences, with relative values of 0.81–0.93. *RAPGEF4*, which encodes Epac2, was relatively higher in Case 1, with values of 0.37–0.75 in Cases 2–4.

Several genes related to regulated exocytosis and vesicle trafficking, *STX1A*, *SYT7*, *SNX19*, and *RAB3A*, tended to be more highly expressed in Case 1. Relative expression values in Cases 2–4 were 0.31–0.69 for *STX1A*, 0.09–0.40 for *SYT7*, and 0.40–0.54 for *SNX19*. In contrast, *SNAP25* was higher in Cases 2 and 3 than in Case 1, indicating that exocytosis-related genes did not change uniformly.

Genes related to integrated stress responses and endoplasmic reticulum stress were generally higher in Cases 2–4. *GDF15* showed marked elevation, with relative expression values of 11.39 in Case 2, 19.43 in Case 3, and 51.63 in Case 4. *ATF4*, *XBP1*, and *EIF2AK3* were also higher in Cases 2–4 than in Case 1, with relative values of 2.04–2.62, 6.23–6.73, and 1.88–2.45, respectively.

Among transcription factors related to β-cell differentiation and functional maintenance, *PDX1*, *PAX6*, and *NEUROD1* were relatively high in Case 1 and lower in Cases 3 and 4. In Case 2, *PDX1* and *NEUROD1* were similar to Case 1, whereas *PAX6* was lower. *HNF1A* was markedly higher in Cases 2–4, with relative expression values of 8.06–42.32, and *HNF1B* was also increased, particularly in Cases 3 and 4.

## Discussion

This study integrated clinical stimulation tests and tumor gene expression profiling in four insulinoma cases with different hypoglycemia patterns. Previous *in vitro* studies have shown that human insulinomas exhibit distinct patterns of insulin secretion and that histologic structure alone is not a sufficient marker of their functional properties (3). The key finding of the present study was that the tumor from the patient with postprandial and stimulus-induced hypoglycemia showed relatively preserved expression of genes involved in glucose sensing, K_ATP_ channel function, incretin/cAMP signaling, exocytosis, and β-cell differentiation. In contrast, insulinomas presenting mainly with fasting hypoglycemia showed higher expression of *HK1* and stress-response genes. Because all tumors were classified as WHO grade 1 with low Ki-67 indices, differences in tumor grade are unlikely to explain the observed transcriptomic and secretory phenotypes. These observations suggest that insulinoma secretory phenotypes may reflect distinct molecular programs.

Case 1 showed marked insulin or C-peptide responses to oral glucose, meal, and glucagon stimulation. This pattern suggests that the tumor cells were not merely releasing insulin autonomously but retained the ability to amplify secretion in response to external stimuli. The relatively high expression of *ABCC8*, *KCNJ11*, *GCK*, *GLP1R*, and *RAPGEF4* supports the possibility that a β-cell-like regulated secretory program was preserved in this tumor. These genes are related to glucose-stimulated insulin secretion, cAMP signaling, insulin granule exocytosis, and β-cell maturity (4, 5, 6, 7, 8, 10, 11). Their preserved expression may explain why Case 1 developed hypoglycemia preferentially after meal or glucose stimulation.

The higher expression of *HK1* in Cases 3 and 4 may be relevant to fasting hypoglycemia. In mature β-cells, glucokinase functions as a glucose sensor because of its relatively high Km, allowing insulin secretion to increase as glucose levels rise (12). Hexokinase 1 has a higher affinity for glucose than for glucokinase and can promote glucose phosphorylation at lower glucose concentrations. In human insulinomas, undue expression of hexokinase 1 in tumor β-cells has been associated with strong insulin secretion at very low glucose concentrations and fasting hypoglycemia (3). Therefore, ectopic or increased *HK1* expression in insulinoma may enhance glycolysis even under low-glucose or fasting conditions, thereby promoting ATP production, K_ATP_ channel closure, membrane depolarization, and insulin secretion. In contrast, Case 2 showed only mild *HK1* elevation and developed hypoglycemia later during fasting. These findings suggest that differences in *HK1* expression may partly contribute to inter-case differences in the time to hypoglycemia during fasting.

Differences in β-cell differentiation state may also contribute to secretory behavior. Case 1 had relatively high expression of *PDX1*, *PAX6*, and *NEUROD1*, which are involved in β-cell identity, insulin gene expression, endocrine differentiation, and functional maturation (13, 14, 15). This pattern suggests a more β-cell-like differentiated state. Cases 3 and 4 showed lower expression of these transcription factors and weaker stimulus responsiveness, which may reflect a less mature or more dedifferentiated tumor cell state. The marked increase in *HNF1A* in Cases 2–4 is notable, because HNF1A is involved in β-cell function and monogenic diabetes (16); however, its relationship with insulinoma differentiation, metabolism, or tumor biology remains unclear and requires further investigation.

Cases 2–4 also showed higher expression of *GDF15*, *ATF4*, *XBP1*, and *EIF2AK3*, suggesting activation of endoplasmic reticulum stress or integrated stress responses. *EIF2AK3* encodes PERK, a major unfolded protein response sensor in β-cells, and PERK-eIF2α signaling induces ATF4-mediated transcriptional responses (17, 18). XBP1 is a key mediator of the IRE1 branch of the unfolded protein response (18). GDF15 has also been implicated in ER stress-induced β-cell apoptosis (19). The association between stress-response gene expression and fasting-type insulinoma in this case series is exploratory. Stress-response gene expression may partly reflect differences in the functional or secretory state of insulinoma cells.

This study has several limitations. First, only four cases were analyzed, and the stimulus-responsive phenotype was represented by a single case. Therefore, the observed gene expression pattern cannot be considered a general feature of all stimulus-responsive insulinomas. Second, mRNA expression changes were not validated at the protein or functional level by immunohistochemistry, western blotting, or ex vivo secretion assays. Third, because bulk tumor RNA was analyzed, the influence of intratumoral cellular heterogeneity could not be excluded. Fourth, although established diabetes was unlikely in Case 2, diabetic-range hyperglycemia was observed during the preoperative OGTT. Therefore, the potential influence of impaired glucose tolerance on stimulation test interpretation and gene expression, including stress-response and β-cell-related genes, cannot be completely excluded. Fifth, differences in systemic metabolic factors, including insulin sensitivity and incretin responses, may have contributed to the clinical phenotypes observed in this small case series. Finally, because this was an exploratory case series, statistical inference was limited. Additional cases and functional validation studies are required.

In conclusion, the insulinoma cases with postprandial and stimulus-responsive hypoglycemia showed marked insulin or C-peptide responses to glucose, meal, and glucagon stimulation, together with relatively preserved expression of genes related to β-cell-like regulated secretion. In contrast, cases presenting mainly with fasting hypoglycemia showed increased expression of *HK1* and stress-response genes. These findings suggest that clinical heterogeneity in insulinoma may reflect differences in regulated secretory capacity, glucose sensing, and cellular stress responses.

## Supplementary materials





## Declaration of interest

The authors declare that there is no conflict of interest that could be perceived as prejudicing the impartiality of the research reported.

## Funding

This research did not receive any specific grant from any funding agency in the public, commercial, or not-for-profit sector.

## Author contribution statement

KS and WO contributed to conceptualization. YO, TM, SA, and YH contributed to methodology. KS, YO, TM, and SA contributed to investigation. KS, YO, and TM contributed to formal analysis and data curation. KS and WO contributed to writing of the original draft. WO contributed to supervision. All authors contributed to review and editing and read and approved the final manuscript.

## Ethics statement

This study was conducted in accordance with the Declaration of Helsinki and was approved by the Ethics Committee of Kobe University Hospital (Approval Number: 79).

## Patient consent statement

Written informed consent was obtained from all patients for participation in this study and for publication of anonymized clinical and research data.

## Data availability

Raw and normalized microarray data have been deposited in the Gene Expression Omnibus (GEO) database under accession number GSE334499. Additional data are available from the corresponding author upon reasonable request.
