# Supplementary material for: Gene expression profiles associated with stimulus-responsive insulin secretion in insulinoma
Source: Endocr Connect. 2026 Jul 15;15(7):e260322. doi: 10.1530/EC-26-0322 (PMC13386152; doi:10.1530/EC-26-0322)
Supplement: Supplementary file 1 [file EC-26-0322_supplementary_table_1.pdf]

Supplementary Table 1. Selective arterial calcium stimulation test (SACST) results

Variable	Case 1	Case 2	Case 3	Case 4
Positive artery/arteries	SMA / GDA	CHA / GDA	Proximal SA	PSPDA
Maximum IRI response	SMA 87-fold; GDA 28-fold	CHA 9-fold; GDA 4-fold	~50-fold	~7-fold
Localization by SACST	Pancreatic head	Pancreatic head	Pancreatic body	Pancreatic head

SMA, superior mesenteric artery; GDA, gastroduodenal artery; CHA, common hepatic artery; SA, splenic artery; PSPDA, posterior superior pancreaticoduodenal artery; IRI, immunoreactive insulin.
