# Supplementary material for: Gene expression profiles associated with stimulus-responsive insulin secretion in insulinoma
Source: Endocr Connect. 2026 Jul 15;15(7):e260322. doi: 10.1530/EC-26-0322 (PMC13386152; doi:10.1530/EC-26-0322)
Supplement: Supplementary file 2 [file EC-26-0322_supplementary_table_2.pdf]

Supplementary Table 2. Preoperative and postoperative oral glucose tolerance test results

Case	Timing	Days after surgery	Parameter	0 min	30 min	60 min	90 min	120 min
Case 1	Preoperative	-	Glucose (mg/dL)	72	114	97	44	29
			IRI (µU/mL)	12	1,483	495	167	76
	Postoperative	17	Glucose (mg/dL)	90	132	115	140	94
			IRI (µU/mL)	6	53	21	35	8
Case 2	Preoperative	-	Glucose (mg/dL)	59	159	156	210	239
			IRI (µU/mL)	4	23	19	34	50
	Postoperative	15	Glucose (mg/dL)	86	145	171	158	154
			IRI (µU/mL)	5	33	10	6	4
Case 3	Preoperative	-	Glucose (mg/dL)	77	121	127	105	100
			IRI (µU/mL)	20	54	66	47	18
	Postoperative	15	Glucose (mg/dL)	111	172	157	120	88
			IRI (µU/mL)	11	55	77	45	24
Case 4	Preoperative	-	Glucose (mg/dL)	77	126	171	155	145
			IRI (µU/mL)	6	6	43	33	39
	Postoperative	49	Glucose (mg/dL)	81	122	102	116	80
			IRI (µU/mL)	3	34	22	17	15

IRI, immunoreactive insulin.
